# Systemic HMGB1 Neutralization Prevents Postoperative Neurocognitive Dysfunction in Aged Rats

**DOI:** 10.3389/fimmu.2016.00441

**Published:** 2016-10-24

**Authors:** Niccolò Terrando, Ting Yang, Xueqin Wang, Jiakai Fang, Mengya Cao, Ulf Andersson, Harris Helena Erlandsson, Wen Ouyang, Jianbin Tong

**Affiliations:** ^1^Department of Anesthesiology, Duke University Medical Center, Durham, NC, USA; ^2^Department of Medicine, Division of Nephrology, Durham VA and Duke University Medical Centers, Durham, NC, USA; ^3^Department of Anesthesiology, Third Xiangya Hospital of Central South University, Changsha, Hunan, China; ^4^Department of Women’s and Children’s Health, Karolinska Institutet, Karolinska University Hospital, Stockholm, Sweden; ^5^Department of Medicine, Karolinska Institutet, Stockholm, Sweden

**Keywords:** aging, HMGB1, inflammation, memory, microglia, surgery

## Abstract

Postoperative neurocognitive disorders are common complications in elderly patients following surgery or critical illness. High mobility group box 1 protein (HMGB1) is rapidly released after tissue trauma and critically involved in response to sterile injury. Herein, we assessed the role of HMGB1 after liver surgery in aged rats and explored the therapeutic potential of a neutralizing anti-HMGB1 monoclonal antibody in a clinically relevant model of postoperative neurocognitive disorders. Nineteen to twenty-two months Sprague-Dawley rats were randomly assigned as: (1) control with saline; (2) surgery, a partial hepatolobectomy under sevoflurane anesthesia and analgesia, + immunoglobulin G as control antibody; (3) surgery + anti-HMGB1. A separate cohort of animals was used to detect His-tagged HMGB1 in the brain. Systemic anti-HMGB1 antibody treatment exerted neuroprotective effects preventing postoperative memory deficits and anxiety in aged rats by preventing surgery-induced reduction of phosphorylated cyclic AMP response element-binding protein in the hippocampus. Although no evident changes in the intracellular distribution of HMGB1 in hippocampal cells were noted after surgery, HMGB1 levels were elevated on day 3 in rat plasma samples. Experiments with tagged HMGB1 further revealed a critical role of systemic HMGB1 to enable an access to the brain and causing microglial activation. Overall, these data demonstrate a pivotal role for systemic HMGB1 in mediating postoperative neuroinflammation. This may have direct implications for common postoperative complications like delirium and postoperative cognitive dysfunction.

## Introduction

Postoperative neurocognitive disorders, encompassing postoperative delirium (POD) and postoperative cognitive dysfunction (POCD), are common complications in elderly patients over 65 years of age following surgery or critical illness (1). Up to 47% of elderly patients experience cognitive dysfunction at hospital discharge and about 10% retain persistent cognitive deficits 3 months after major surgery (2). Occurrence of these postoperative complications significantly associate with increased morbidity and mortality, greater length of hospital stay, increased costs, and decreased life independence (3). These outcomes, combined with the constant increase in the geriatric population, render postoperative neurocognitive disorders a significant complication that currently lacks defined mechanisms and treatments.

It is well appreciated that sterile injury, including surgical trauma, triggers a milieu of factors that ultimately contribute to the inflammatory process (4). Damage-associated molecular pattern molecules (DAMPs), in particular high mobility group box 1 (HMGB1), are rapidly released after tissue trauma and mediate immune cell recruitment and activation, cytokine release, and cell death (5). This molecule is highly preserved through evolution and is 99% identical between mammals (6). HMGB1 release has been described in preclinical models of cognitive decline (7–9). However, its contribution to postoperative neuroinflammation remains unclear. Increased levels of HMGB1 after surgery have been related to compromising the blood–brain barrier (BBB) and subsequent neuroinflammation (10, 11). Recently, changes in serum HMGB1 and other pro-inflammatory cytokines were positively associated with human POCD after gastrointestinal surgery in elderly patients (12).

Based on the above, we assessed the role of HMGB1 after liver surgery in aged rats and the therapeutic potential of a neutralizing anti-HMGB1 monoclonal antibody (mAb) as a mean to prevent postoperative neurocognitive disorders. Herein, we show a prominent role for systemic HMGB1 signaling to mediate neuroinflammation and neurotoxicity. Anti-HMGB1 mAb treatment increased phosphorylation of cyclic AMP response element binding (p-CREB) in the hippocampus after surgery, suggesting that anti-HMGB1 exerts neuroprotective effects in aged rats. These effects on neuroinflammation and synaptic plasticity may inform on pathophysiological mechanisms of delirium and other postoperative neurocognitive disorders appearing after surgery.

## Materials and Methods

### Animals

Experiments were performed in accordance with the guidelines for experimental animal use of the Central South University. The protocol [LLSC(LA)2015-003] was approved by the ethics committee of the third Xiangya Hospital of Central South University. Aged female Sprague-Dawley (SD) rats (19–22 months, 450–600 g) were purchased from Central South University (China). Rats had surgery in the diestrus phase when estrogen levels are at their minimum. All rats were housed in standard cages with free access to food and water.

### Drug Administration

Aged rats were randomly divided into 3 groups: (1) control with intravenous (i.v.) saline only; (2) surgery + immunoglobulin (IgG) (S + IgG) (1 mg/kg i.v.); (3) surgery + anti-HMGB1 (S + anti-HMGB1) (1 mg/kg i.v.). Treatments were given *via* tail vein immediately before surgical incision and 6 h after surgery; dosage and timing was based on Okuma et al. (13). Anti-HMGB1 antibody (2G7, mouse IgG2b) was supplied by Dr. H.E. Harris’s laboratory, Stockholm, Sweden. This antibody has been extensively characterized previously with respect to its HMGB1 neutralizing activity in *in vitro* and *in vivo* studies (9, 14–18). The 2G7 anti-HMGB1 mAb neutralizes both HMGB1-induced cytokine/chemokine release and chemotactic activities. Mouse IgG2b (Sigma, M1395-5MG) was used as an isotype control. A separate cohort of rats was divided into 4 groups: (1) control saline alone; (2) surgery with saline; (3) surgery with Histidine (His)-tagged HMGB1 (1 mg/kg); (4) His-tagged HMGB1 only (1 mg/kg). His-tagged HMGB1 (Sigma, cat. Number 4652) or saline was given *via* tail vein right before surgical incision.

### Partial Hepatolobectomy

Rats were rapidly induced with 5% sevoflurane anesthesia (Maruishi Pharmaceutical Co., Ltd., Japan) with high flow of oxygen (6 L/min). As each rat achieved loss of righting reflex, it was intubated with a 14G catheter and anesthesia maintained with continuous delivery of 3.5–4.5% sevoflurane mixed with oxygen (80–85%). The gas was monitored and analyzed by a multi-function monitor (Datex-Ohmeda, Helsinki, Finland); respiratory rate (*R*), PetCO_2_, FiO_2_, and Fi_Sev_ were continuously recorded. The depth of anesthesia was modulated according to the *R* (30–50/min) and the body movement of the rats. The partial hepatolobectomy was performed as previously described with some modifications (19). Briefly, an incision about 2 cm long was made below the xyphoid; the left lobe of liver was carefully isolated, ligated, and then removed. Finally, muscles and skin were closed with sterile sutures and subcutaneous tissue infiltration with 0.2 mL of 0.25% bupivacaine was administered for the purpose of local postoperative analgesia. Animals were then allowed to recover for further testing.

### Behavioral Tests

#### Barnes Maze

Rats were tested with a protocol previously described (20). Briefly, rats were trained to locate the escaping hole on a Barnes maze four times/day on postoperative days 1–4 (3 min/trial and 15 min between each trial). The number of incorrect hole investigation (termed error) during each trial were recorded. The platform surface was cleaned with 75% ethanol before each trial in order to remove odor cues.

#### Open Field

Open field test was used to evaluate the anxiety level of the animal. A rat was placed directly into the center of the open field (100 cm × 100 cm × 48 cm, length × width × height). Movement of the rat in the open field was recorded by a digital camera during the 5-min testing session. The total square crossings, time spent in the central area and the percentage of square crossings in the central area to the total square crossings, were counted.

### Immunostaining

Rats were terminated with chloral hydrate (10%) and perfused transcardially with ice cold 0.01M phosphate-buffered saline (PBS). The brain was rapidly dissected; one hemisphere was used for immunostaining and the other for western blot. The hemisphere used for immunostaining was postfixed in 4% paraformaldehyde overnight at 4°C. After dehydrating with 15% and 30% sucrose, the brains were embedded in OCT (SAKURA Tissue-Tek, USA), and cryostat transverse sections of the brain (20 μm) were obtained. Four sections of hippocampus were randomly picked from 4 sets of serial sections from each rat at −3.6 to −4.16 mm anteroposterior to the bregma for immunostaining. Sections were washed three times in 0.01M PBS and incubated in 3% H_2_O_2_ for 10 min. After three washes, sections were blocked with 5% BSA in 0.01M PBS plus 0.1% Triton X-100 for 1 h at room temperature and then incubated with primary antibodies (1:1000, Iba-1, HMGB1, p-CREB) at 4°C overnight. Full details on the antibodies used are presented in Table 1. After washing three times in 0.01M PBS, the sections were incubated with secondary antibodies (Biotinylated Goat Anti-Rabbit IgG, 1:200, Vector, USA) for 1 h at room temperature, subsequently washed three times in 0.01M PBS and finally covered with permount containing DAPI (Vector, USA, H-1200). For each staining, pictures of Cornu Ammonis (CA)1 and Dentate Gyrus (DG) areas were taken under the same magnification (40× objective lens) and constant light intensity with a microscope (DS-Ri1, Nikon, Japan) by an author blinded to treatments.

**Table 1:** **List of antibodies used in the study**.

Antibody	Host	Antigen	Supplier/cat. num.	Concentration
HMGB1	Rabbit/monoclonal	Synthetic peptide corresponding to Human HMGB1 aa 150 to the C-terminus	Abcam, ab79823	1:1000
p-CREB	Rabbit/monoclonal	Synthetic phoshopeptide corresponding to residues surrounding Serine 133 of human CREB	Abcam, ab32096	1:1000
Iba1	Rabbit/polyclonal	Synthetic peptide corresponding to C-terminus of Iba1	Wako, 019-19741	1:1000
NR2A	Rabbit/monoclonal	Synthetic peptide corresponding to Human NMDAR2A	Epitomics, 3916-1	1:2000
NR2B	Rabbit/polyclonal	Synthetic peptide conjugated to KLH derived from within residues 1450 to the C-terminus of Rat NMDAR2B	Abcam, ab65783	1:1000
His	Mouse/monoclonal		Sigma, H1029	1:5000
GAPDH	Rabbit/polyclonal	full-length GAPDH of human origin	Proteintech, 10494-1-AP	1:2000

### Western Blot

Western blotting was used to assess the expression of *N*-methyl-d-aspartate receptor (NMDAR) subunits NR2A and NR2B, HMGB1, and GAPDH in the hippocampus. Briefly, frozen hippocampus was homogenized in lysis buffer containing protease inhibitors cocktails (Roche, Germany, cat num P8340) and phenylmethanesulfonylfluoride (PMSF, Sigma, USA, cat num p7626). The quantity of protein of samples was determined using a BCA protein assay kit (CWBio, China) according to the manufacturer’s instructions. Equal amounts of protein samples were separated by sodium dodecyl sulfate polyacrylamide gel electrophoresis (SDS-PAGE) and transferred to polyvinylidene fluoride membranes. Membranes were blocked with 10% skim milk in TBST buffer for 1 h and then incubated with primary antibodies, HMGB1 (1:1000 Abcam, USA), NR2A (1:2000 Epitomics, USA), NR2B (1:1000 Abcam, USA), GAPDH (1:2000 Proteintech, China), and GAPDH (1:2000 Proteintech, China), anti-His antibody (1:5000 Sigma) overnight at 4°C (Table 1). After three washes, membranes were incubated with the secondary antibodies (Goat Anti-Rabbit IgG, HRP Conjugated, 1:2000, CWBIO, China, catalog number CW0103, Goat Anti-Mouse IgG, HRP Conjugated, 1:2000, CWBIO, China, catalog number CW0102) at room temperature for 2 h. Finally, visualization of the proteins was accomplished by enhanced chemiluminescence detection kit (Pierce; Thermo Scientific, Shanghai, China), and the intensity of each band was quantified by densitometry. Relative expression levels of protein were normalized by the ratio of target protein (HMGB1, NR2A, and NR2B) to GAPDH.

### Enzyme-Linked Immunosorbent Assay

Under anesthesia, blood was collected from the right auricle in EDTA coated tubes and then centrifuged for 10 min at 3000 rpm. Plasma was collected and frozen at −80°C for further analyses. Human plasma was collected under an approved protocol by the Ethics Committee of the Third Xiangya Hospital of Central South University (protocol number: 2015-S165). Six patients identified with POD by The Confusion Assessment Method (CAM) (21) daily testing were compared to age-matched surgical controls (non-delirious). Blood was centrifuged at 3000 rpm for 10 min at room temperature, and the plasma samples were immediately frozen at −80°C. Concentration of HMGB1 in plasma was measured using ELISA (IBL International, Germany, catalog number ST51011) according to the manufacturer’s protocol.

### Statistical Analysis

Data are shown as mean ± SEM. Two-way repeated measures ANOVA was used to analyze the data from Barnes Maze test. ANOVA was used to analyze the data from open field test, Western blot, and immunostaining. Bonferroni Multiple Comparison Test was performed to compare selected groups when ANOVA showed significance. Statistical analysis was performed using Prism 5 (Graph Pad Software Inc., La Jolla, CA, USA). Significance was set at *p* < 0.05.

## Results

### Blocking HMGB1 Improves Postoperative Cognitive Decline and Anxiety in Aged Rats

**Figure 1:**
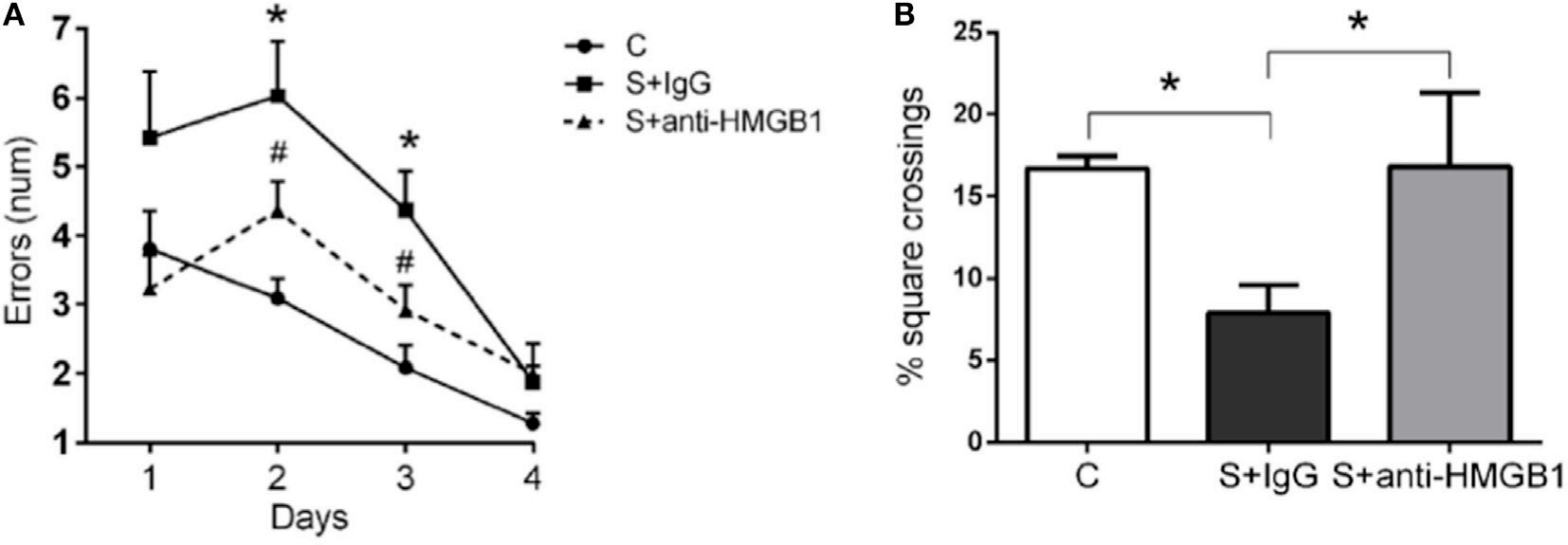
**Anti-HMGB1 improves memory dysfunction and anxiety in aged rats**. Spatial memory was evaluated in the Barnes maze. Rats that had undergone liver surgery had significant memory impairments as detected by error numbers in reaching the correct target box **(A)**. Treatment with anti-HMGB1 mAb significantly improved postoperative memory dysfunction on both days 2 and 3 after surgery. **(B)** Anxiety was measured in an open field on postoperative day 3. Treatment with anti-HMGB1 mAb prevented postoperative anxiety as compared to the S + IgG group. Results are expressed as mean ± SEM (*n* = 12). *p < 0.05 vs. C; #*p* < 0.05 vs. S + IgG group by two-way repeated measures ANOVA for Barnes Maze and one-way ANOVA for open field. Abbreviations: C, control; S, surgery.

All rats were included in the study protocol, and no mortality was reported after the surgical procedure. We evaluated learning and memory by error number in the Barnes maze and detected levels of anxiety in the open field. In this test, repeated measures ANOVA showed that the error number was significantly affected by treatment [*F*_(2,69)_ = 5.73, *p* = 0.01], measure time [*F*_(3,69)_ = 36.79, *p* < 0.001], and the interaction of treatment and measure time [*F*_(6,69)_ = 3.41, *p* = 0.005]. Compared to the control group, the errors of the surgery group treated with control antibodies (S + IgG group) were significantly increased on days 2 and 3 after surgery, indicating an impairment in learning and memory of aged rats after surgery (*p* = 0.001, respectively) (Figure 1A). Compared to the S + IgG group, errors in S + anti-HMGB1 group were significantly decreased on days 2 (*p* = 0.035) and 3 after surgery (*p* = 0.023), denoting a protective effect by perioperative anti-HMGB1 treatment (Figure 1A).

In addition, in the open field test, the percentage of square crossing in the central area in the S + IgG group was significantly lower than that of control group on day 3 after surgery (7.88 ± 1.74 vs. 16.68 ± 0.79%, *p* < 0.05) (Figure 1B). Percentage square crossing in the central area in S + anti-HMGB1group significantly increased relative to the S + IgG group (16.79 ± 4.53 vs. 7.88 ± 1.74%, *p* < 0.05) (Figure 1B).

### Effects of Anti-HMGB1 on Memory Marker p-CREB

**Figure 2:**
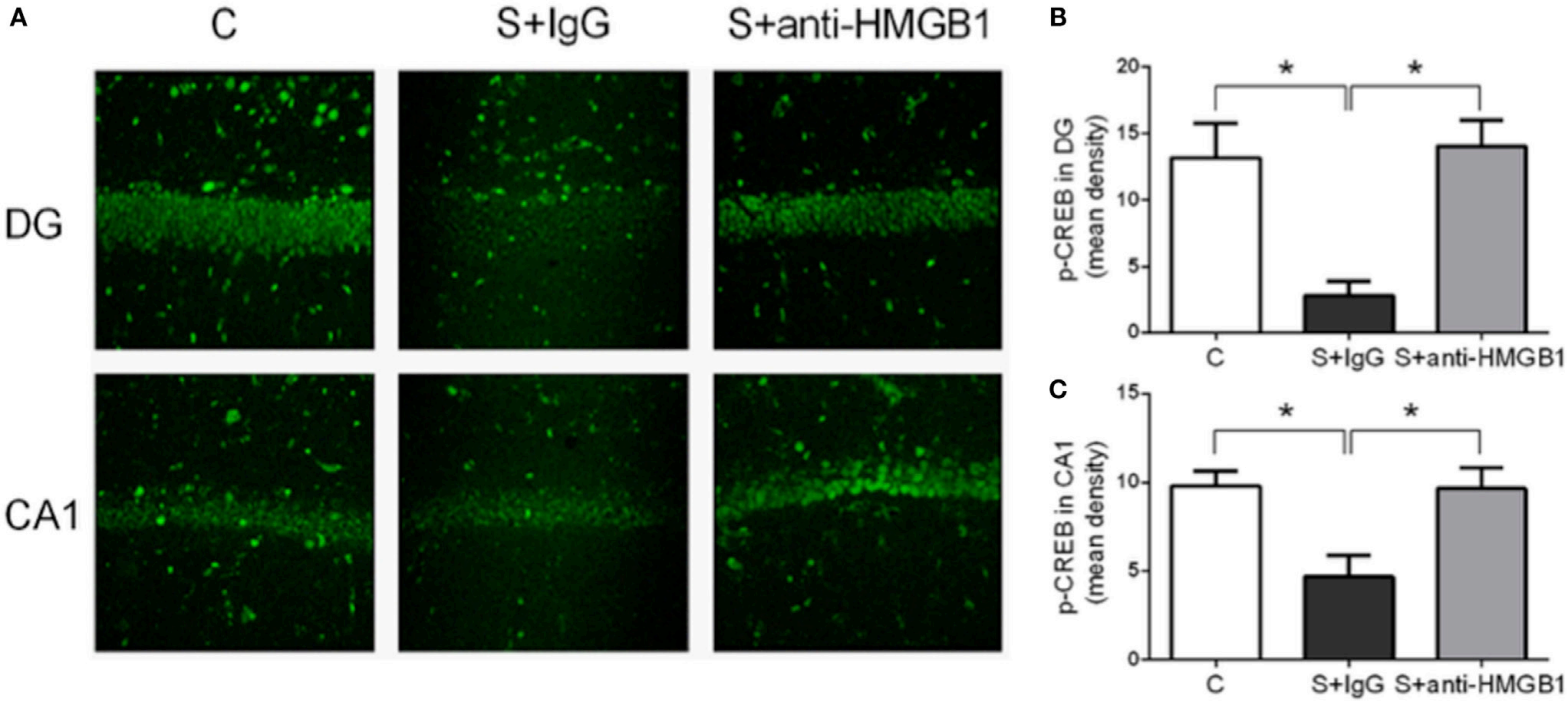
**Modulation of p-CREB by anti-HMGB1**. Representative p-CREB immunofluorescence in the hippocampus, DG and CA1 areas, on postoperative day 3 **(A)**. Expression of p-CREB was reduced after surgery (S + IgG), and anti-HMGB1 treatment was effective in rescuing the expression back to levels similar to the levels of control animals. **(B,C)** show relative mean optical density for p-CREB staining in DG and CA1. Results are expressed as mean ± SEM (*n* = 5). Abbreviations: C, control; S, surgery. Scale bar = 50 μm.

To further investigate the mechanisms of anti-HMGB1 in preventing surgery-induced memory decline, we assessed the transcription factor CREB, which is closely involved in synaptic plasticity and memory function (22). Compared to the control group, the level of p-CREB in the DG area of the S + IgG group was distinctly decreased on day 3 (13.19 ± 1.05 vs. 2.85 ± 0.43, *p* < 0.05) (Figures 2A,B). Rats treated with anti-HMGB1 mAb had significantly higher p-CREB in the DG and CA1 areas than the S + IgG group (14.04 ± 0.80 vs. 2.85 ± 0.43 and 9.66 ± 0.47 vs. 4.68 ± 0.50, *p* < 0.05, respectively), which was similar to control rats (Figures 2B,C).

**Figure 3:**
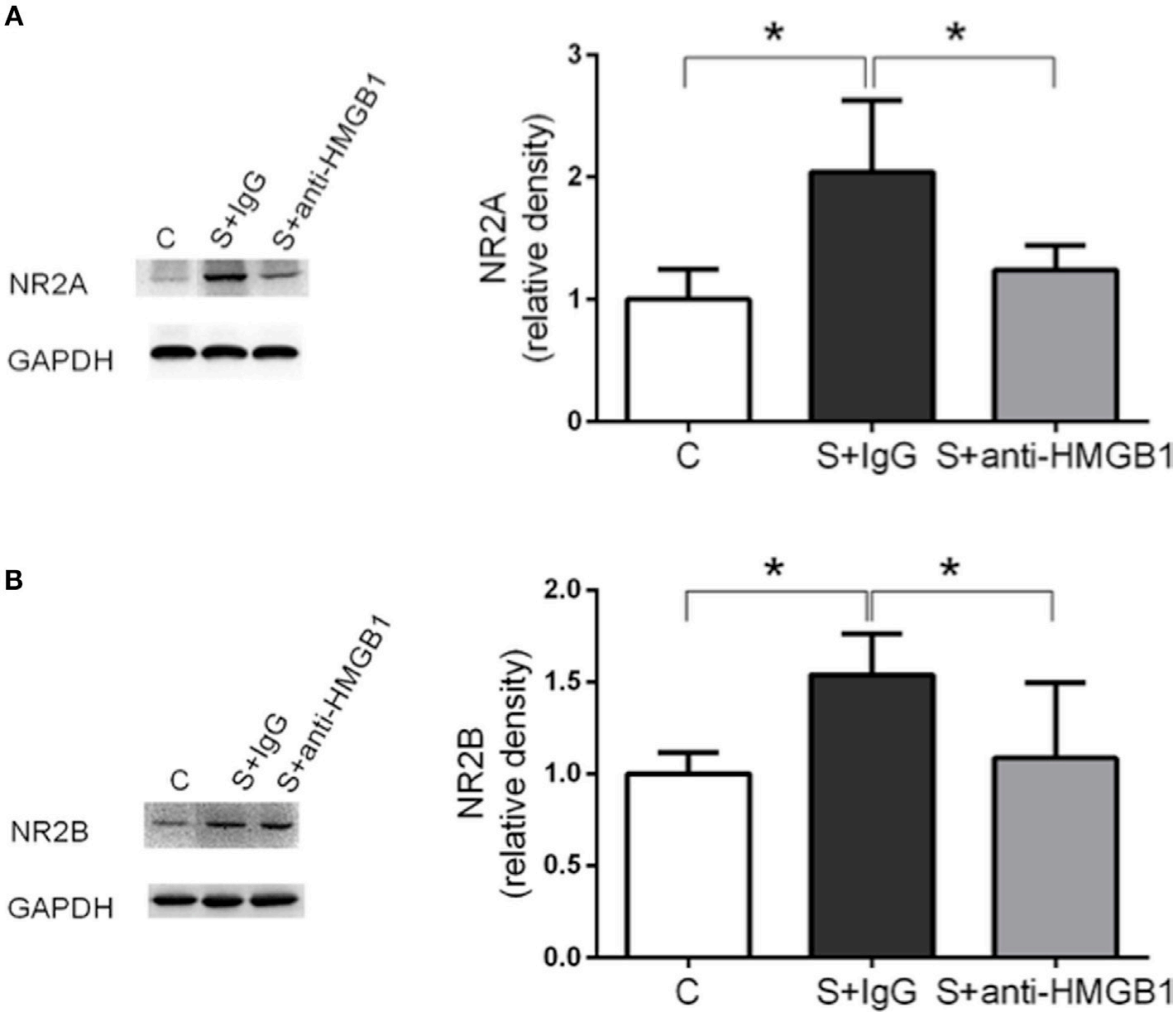
**Effects of anti-HMGB1 on NMDAR subunits**. Expression of NR2A **(A)** and NR2B **(B)** was assessed in the hippocampus on day 3. Immunoblots and quantifications are shown describing an increase in NR2A and NR2B levels after surgery that was restored almost to baseline by anti-HMGB1 mAb treatment. Results are expressed as mean ± SEM (*n* = 5). *p < 0.05 by one-way ANOVA. Abbreviations: C, control; S, surgery. NR2A/B: *N*-methyl-d-aspartate receptor subunit 2A/B.

We also assessed NMDAR expression in hippocampal lysate. Compared to control, expression of NMDAR subunits NR2A and NR2B was increased at day 3 after surgery (1.00 ± 0.09 vs. 2.04 ± 0.21 and 1.00 ± 0.04 vs. 1.54 ± 0.08, *p* < 0.05, respectively) (Figures 3A,B). Treatment with anti-HMGB1 mAb significantly reduced the upregulation, with levels returning to baseline at day 3 as compared to the S + IgG group (2.04 ± 0.21 vs. 1.24 ± 0.07 and 1.54 ± 0.08 vs. 1.09 ± 0.15, *p* < 0.05, respectively) (Figure 3).

### Microglia Activation Is Attenuated by Anti-HMGB1 Treatment

**Figure 4:**
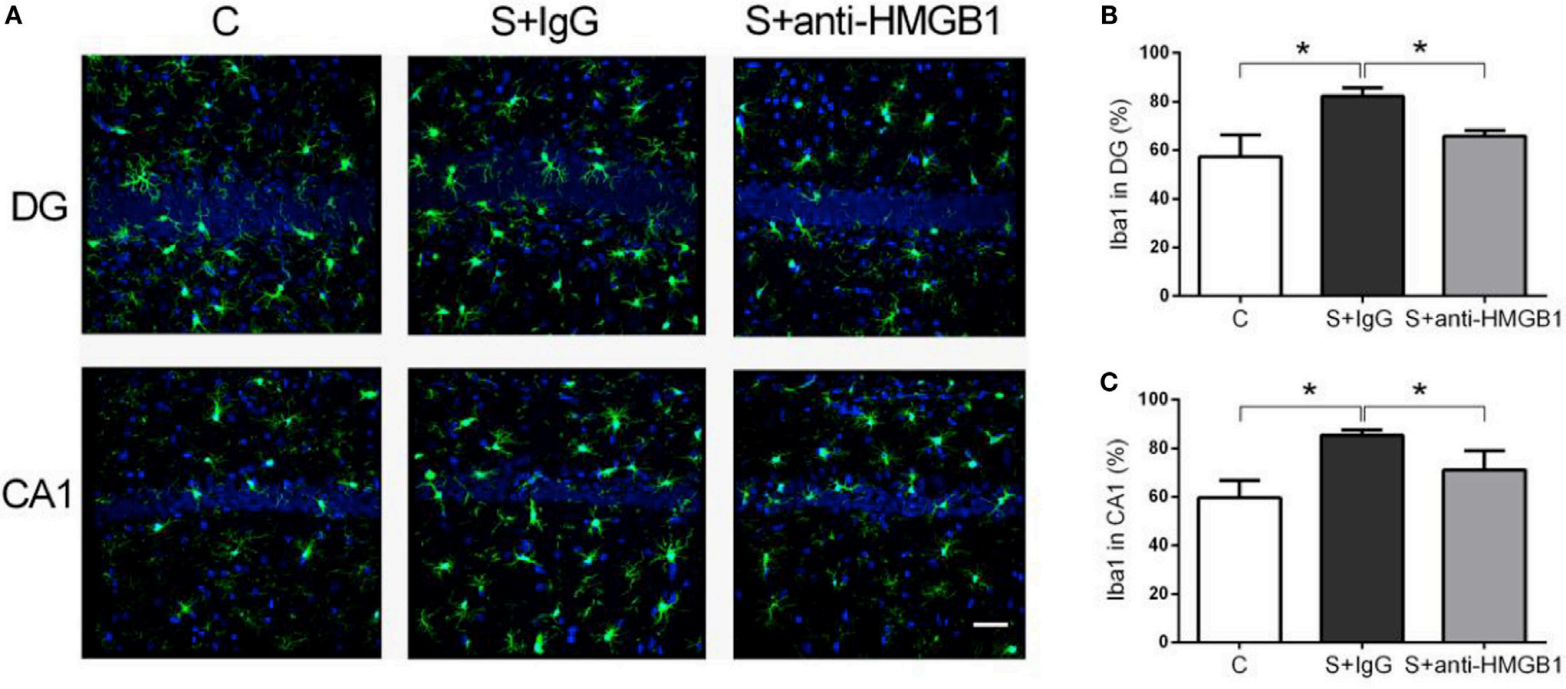
**Microglia activation after surgery and effects of anti-HMGB1**. Neuroinflammation was assessed by Iba-1 immunofluorescence on postoperative day 3. Photomicrographs from DG and CA1 areas of the hippocampus are shown **(A)**. Surgery activated microglia as noted by morphological changes on day 3, including enlargement of cell bodies, which was attenuated by anti-HMGB1 mAb treatment. **(B,C)** show the quantification of Iba-1 immunofluorescence in DG and CA1, respectively. Results are expressed as mean ± SEM (*n* = 5). *p < 0.05 by one-way ANOVA. Abbreviations: C, control; S, surgery. Scale bar = 50 μm.

Neuroinflammation and microglia activation have been implicated in the pathophysiology of cognitive decline (23). Compared to control, the percentage of activated microglia, defined by pleomorphic and bigger cell body with de-ramification and shortening of cell processes, was significantly higher in the CA1 and DG hippocampal areas after surgery (59.64 ± 2.54 vs. 85.30 ± 0.90 and 57.23 ± 3.25 vs. 82.25 ± 1.36, *p* < 0.05, respectively) (Figure 4). Notably, treatment with anti-HMGB1 mAb decreased microglia activation in CA1 and DG at day 3 after surgery (71.10 ± 3.30 vs. 85.30 ± 0.90 and 65.92 ± 0.95 vs. 82.25 ± 1.36, *p* < 0.05, respectively) (Figure 4).

### Peripheral HMGB1 Contributes to Postoperative Neuroinflammation

**Figure 5:**
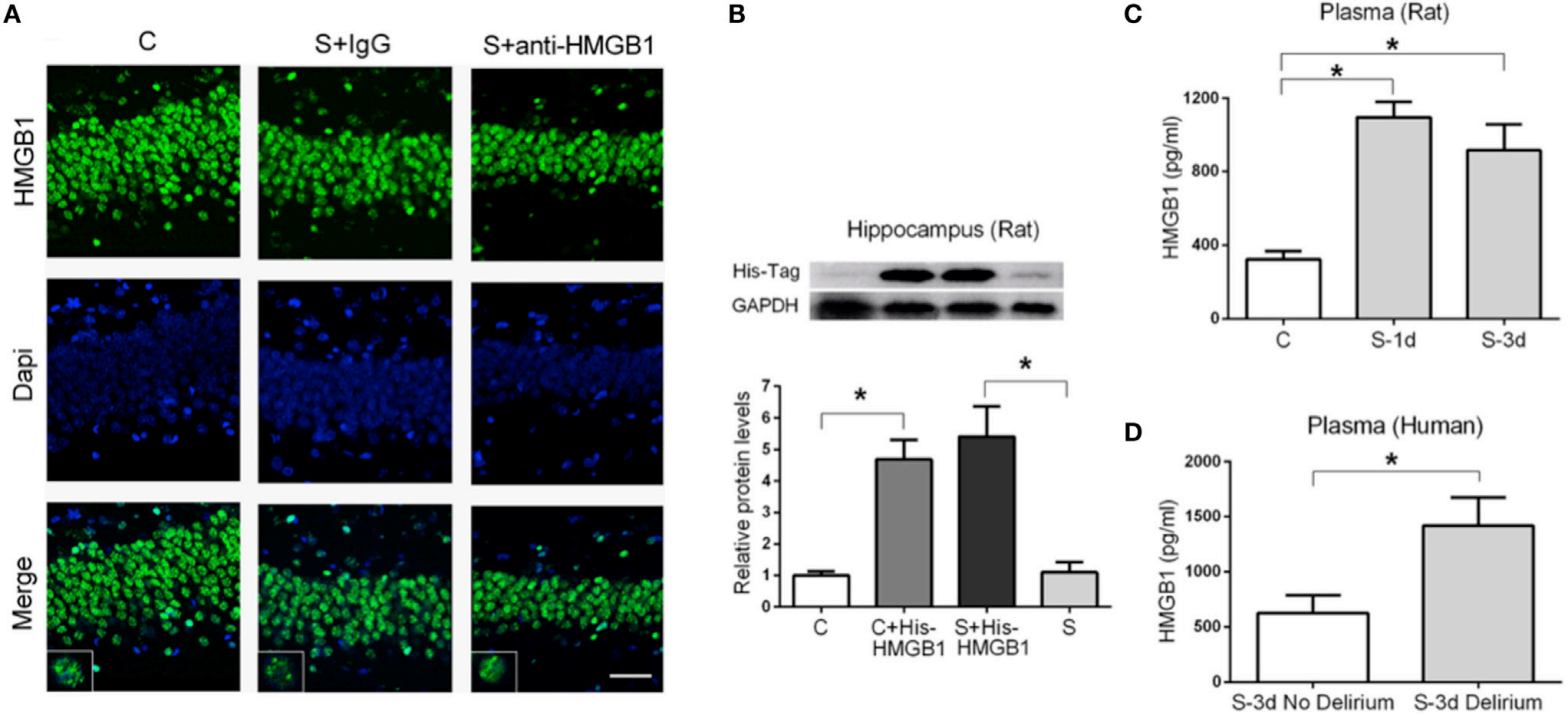
**Expression of HMGB1 in the hippocampus and plasma after surgery**. HMGB1 localization in the hippocampus was assessed on postoperative day 3 **(A)**. No evident changes regarding nuclear translocation or extracellular HMGB1 were detected after surgery (S + IgG) or in S + anti-HMGB1 groups. Images and insets are representative of the hippocampal DG area. We used His-tagged HMGB1 to detect its presence in the CNS after tail vein administration **(B)**. Representative immunoblot and quantification show the presence of His-tagged HMGB1 in the hippocampus of both control (C + His-tagged HMGB1) and operated rats (S + His-tagged HMGB1). Systemic levels of HMGB1 in rats **(C)** and human **(D)** plasma were measured by ELISA on days 1 and 3 after surgery. Results are expressed as mean ± SEM. Scale bar = 50 μm.

To characterize the role of HMGB1 in neuroinflammation after surgery, we measured the cellular distribution and expression of HMGB1 in the hippocampus. Nuclear translocation of HMGB1 to the cytoplasm has been reported as a critical pathological mechanism in a number of different conditions (24, 25). Notably, we found no evident change in cellular localization of HMGB1 in the hippocampus after surgery (Figure 5A).

This prompted us to evaluate if peripheral HMGB1 was responsible for this neuroinflammatory response. We used His-tagged HMGB1 injected into the tail vein of aged rats to detect blood-derived HMGB1 and found a significant increase in His-tagged HMGB1 in the hippocampus of both control and surgery groups (1.00 ± 0.13 vs. 4.69 ± 0.61 and 5.41 ± 0.95 vs. 1.10 ± 0.32, *p* < 0.05, respectively) (Figure 5B) suggesting that peripheral HMGB1 could directly enter into the CNS. Plasma levels of HMGB1 were significantly increased in aged rats at days 1 and 3 after surgery (322.20 ± 45.25 vs. 1098.00 ± 82.64 and 322.20 ± 45.25 vs. 918.30 ± 140.80, *p* < 0.05, respectively) (Figure 5C). We also evaluated human plasma levels of HMGB1 from patients diagnosed with POD on day 3 and found a significant elevation compared to age-matched surgical subjects without delirium (627.1 ± 160.2 vs. 1418 ± 258.4, *p* < 0.05) (Figure 5D).

## Discussion

The current study aimed at investigating the role of HMGB1 signaling after surgery and the therapeutic potential of neutralizing anti-HMGB1 mAbs to prevent surgery-induced memory dysfunction. Our data indicate a key role for peripherally released HMGB1 causing neuroinflammation and memory dysfunction after abdominal surgery. Treatment with a neutralizing anti-HMGB1 mAb reduced microglial activation and prevented synaptic plasticity dysfunction. Herein, we uncovered a novel mechanism for HMGB1 in regulating p-CREB and synaptic plasticity after surgery.

HMGB1 is a multifunctional molecule with a critical role in sterile injury, including surgical manipulations (26). Passive and active release of HMGB1 may occur after injury: passive release occurs instantaneously as a result of disruption of cellular integrity and tissue necrosis; active secretion is a slower process due to the interaction with other cellular products (5, 27, 28). Release of HMGB1 as well as other DAMPs has been reported after surgery, with levels rapidly increasing within 30 min after orthopedic injury (10), suggesting that the release of HMGB1 may be critical in initiating an inflammatory cascade leading to CNS dysfunction. Furthermore, increased systemic levels of HMGB1 have been reported in elderly patients after gastrointestinal surgery, and these levels correlated with the development of POCD (12). Vacas et al. (29) showed a single dose of HMGB1 mAb effectively reduce circulating levels of HMGB1 and IL-6 after orthopedic surgery in younger mice. In our model, we found a more prolonged upregulation of systemic HMGB1. This may be related to a direct surgical insult to the liver, which contains a large pool of HMGB1, and greater cellular senescence promoting age-associated inflammation (30, 31). Thus, we treated animals twice with mAb to prevent both the initial, passive, release of HMGB1 from the direct tissue trauma, and the secondary increase due to the ensuing inflammatory response.

The mechanism whereby peripheral inflammation contributes to neuroinflammation, neurodegeneration, and cognitive dysfunction remains unclear. Intracellular translocation of HMGB1 from the nucleus to the cytoplasm is critical for extracellular release by a non-classical secretory mechanism (24, 32). In our model, we did not observe evident translocation of HMGB1 from the nucleus to the cytoplasm in the CNS. The subcellular localization of HMGB1 in the studied hippocampal areas after surgery was similar to normal expression in the rat brain (33). It is possible that the protective effects in our model are mediated by an overall dampening of systemic DAMPs and cytokines, which in turn prevent endothelial dysfunction and neuroinflammation, and also a direct CNS effect or vagal regulation (34). Neuroinflammation has been reported in several models of POCD (10, 11, 35), and here we could confirm microglia activation in the hippocampal formation after surgery. The important discovery of the present study is that neuroinflammation was reduced by treatment with anti-HMGB1 mAb. Notably, HMGB1 is necessary and sufficient to trigger memory dysfunction (29), microglia activation (36), and prime the immune system (37) as demonstrated by administering recombinant HMGB1 in otherwise healthy animals. Thus, in POCD models preventing systemic HMGB1 increase may be critical in limiting BBB opening (11, 38, 39), subsequent monocyte chemoattractant protein (MCP-1) expression in the hippocampus (29), microglial activation, and memory deficits. The role of the systemic milieu in triggering CNS complications after surgery is further demonstrated by the experiments with His-tagged HMGB1, suggesting that peripheral HMGB1 can directly access the brain parenchyma of aged rats.

In this study, we measured CREB expression in the hippocampus, which is critical in memory and synaptic plasticity (22). Phosphorylated CREB was significantly decreased 3 days after surgery and treatment with anti-HMGB1 mAb reversed this impairment in the hippocampus. Since CREB is implicated in hippocampal-dependent memory function and synaptic plasticity, the rescuing effect of the anti-HMGB1 mAb treatment is consistent with the improved behavioral outcomes demonstrated in this study. Although we can describe a novel effect of anti-HMGB1 mAb to modulate neuroplasticity, the effects of peripheral surgery on neuronal function and p-CREB regulation require further elucidation. POCD has been associated with changes in synaptic plasticity, including long-term potentiation (LTP), and this may be dependent on the neuroinflammatory response after trauma (40). In this study, we found changes in NMDAR expression after surgery, which was restored by anti-HMGB1 mAb treatment. NR2A and NR2B are critical for sustaining LTP and are prominently expressed in hippocampal pyramidal excitatory cells (41). The increase in NR2A and NR2B after surgery may relate to acute neurotoxicity as higher expression of NR2A in the hippocampus has been associated with poorer cognitive outcomes (42). Furthermore, HMGB1 has been demonstrated as an excitatory and neurotoxic signaling molecule in the CNS (43) and exerts a prominent role in conditions like epilepsy (44).

The anti-inflammatory effects of HMGB1 neutralization have been described in several models, including POCD (7–9, 29). Here, we provide additional evidence for HMGB1 modulation of neuronal molecules of relevance to synaptic plasticity and memory function.

In the present study, we have not determined the redox states of HMGB1. This is critical to a better understating of the role of HMGB1 signaling after trauma as posttranslational modifications regulate receptor usage and thus functionality (45). The HMGB1-receptors TLR4 and RAGE have been demonstrated to be upregulated in POCD models (11, 35). No HMGB1 redox isoform restriction is known for RAGE signaling, while disulfide-HMGB1 is required for interaction with TLR4 and TLR4-mediated neuroinflammation and disruption of memory-related signaling in the hippocampus. Moreover, TLR4 activation modulates NMDAR subunits through HMGB1 signaling (46), suggesting that neuroinflammation may be critical in mediating the cognitive deficits.

**Figure 6:**
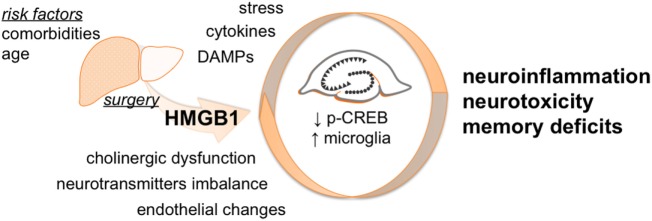
**Proposed working model**. Surgery triggers peripheral HMGB1 release that can gain access to the CNS contributing to neuroinflammation. The effects of HMGB1 in the CNS are not limited to microglia activation but also involve modulation of p-CREB expression in the hippocampus. Overall, this acute neurotoxicity leads to behavioral deficits that are effectively attenuated by selective anti-HMGB1 mAb treatment.

Postoperative neurocognitive disorders are multifactorial conditions with several factors contributing to overall changes in mental status (Figure 6). Recent evidence suggests women are at greater risk for cognitive decline after surgery than men (47, 48). Although we did not include age-matched males or younger females for comparison in this study, several studies have focused on POCD using males and younger rodent models. Herein, we attempted to bridge a critical gap in translation by applying a clinically relevant model to study the therapeutic effects of HMGB1 mAb in aged females. Given HMGB1 is a protein highly conserved across species (6), these findings may have implications beyond specific sex, age, and strain differences.

In conclusion, blocking HMGB1 during the perioperative period attenuated postoperative neuroinflammation and memory dysfunction. Based on these findings, we propose a key role for HMGB1 in mediating POD and provide new evidence for a protective role of neutralizing HMGB1 treatment in the perioperative setting.

## Author Contributions

NT, TY, WO, and JT conceived of the study, analyzed the data, and wrote the manuscript. XW, JF, and MC performed the research. UA and HEH participated in the design of the study and contributed key reagents. All authors read and approved the final manuscript.

## Conflict of Interest Statement

The authors declare that the research was conducted in the absence of any commercial or financial relationships that could be construed as a potential conflict of interest.
