# Supplementary figures and images for: Systemic HMGB1 Neutralization Prevents Postoperative Neurocognitive Dysfunction in Aged Rats
Source: Front Immunol. 2016 Oct 24;7:441. doi: 10.3389/fimmu.2016.00441 (PMC5075578; doi:10.3389/fimmu.2016.00441)

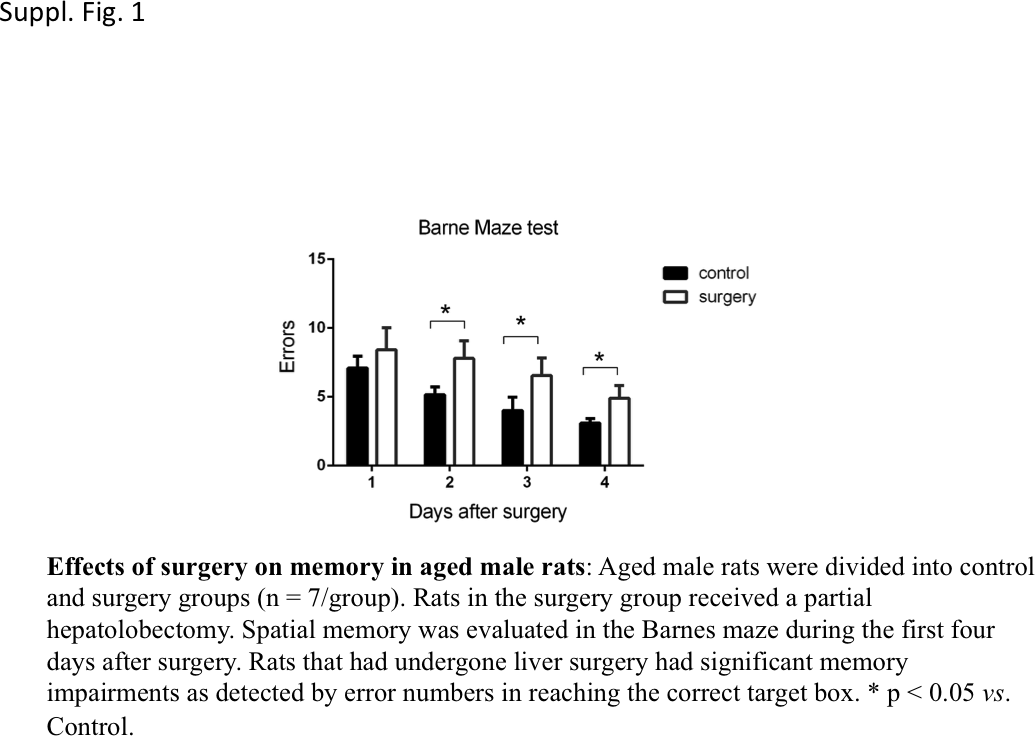

Supplement: Supplementary file 1 [file Image_1.tif]
